# Supplementary material for: Structure and pro-toxic mechanism of the human Hsp90/PPIase/Tau complex
Source: Nat Commun. 2018 Oct 31;9:4532. doi: 10.1038/s41467-018-06880-0 (PMC6208366; doi:10.1038/s41467-018-06880-0)
Supplement: Supplementary file 1 — Supplementary Information [file 41467_2018_6880_MOESM1_ESM.pdf]

Structure and pro-toxic mechanism of the human Hsp90/PPIase/Tau complex

J. Oroz, et al.

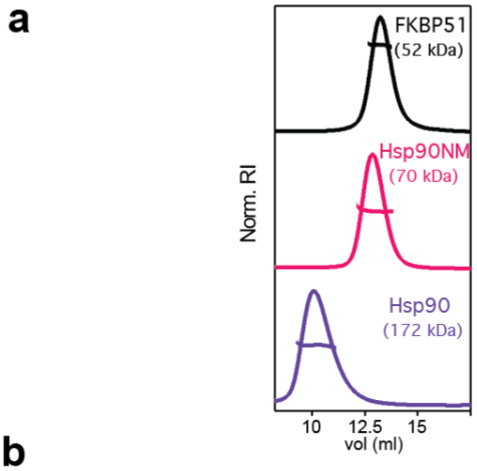

b

Protein	Rg [nm]	Dmax [nm]
Hsp90	6.73 ± 0.10	23.92 ± 0.72
Hsp90 [ADP]	6.34 ± 0.16	20.94 ± 0.52
Hsp90 [AMP.PNP]	6.76 ± 0.15	21.61 ± 0.38
Hsp90:Tau (1:1)	7.39 ± 0.27	24.74 ± 0.27
Hsp90:FKBP51 (1:1)	7.30 ± 0.22	24.36 ± 0.59
Hsp90:FKBP51 (1:1)[ADP]	7.05 ± 0.26	22.50 ± 1.09
Hsp90:FKBP51 (1:1) [AMP.PNP]	6.95 ± 0.30	22.40 ± 1.09
Hsp90:Tau:FKBP51 (1:1:1)	7.52 ± 0.27	25.10 ± 0.38
Hsp90:Tau:FKBP51 (1:1:1)[ADP]	7.37 ± 0.19	23.31 ± 0.09
Hsp90:Tau:FKBP51(1:1:1)[AMP.PNP]	7.15 ± 0.35	23.69 ± 0.78
Hsp90NM	3.82 ± 0.05	13.07 ± 0.20
Tau	6.69 ± 0.17	22.39 ± 1.10
FKBP51	4.96 ± 0.06	15.89 ± 0.49

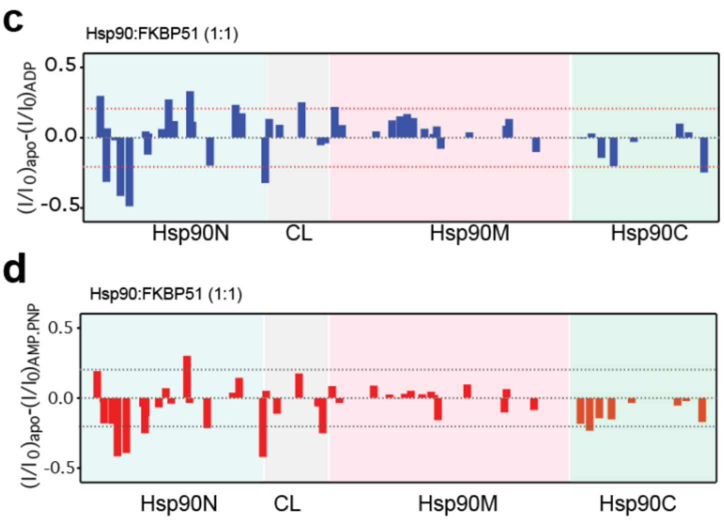

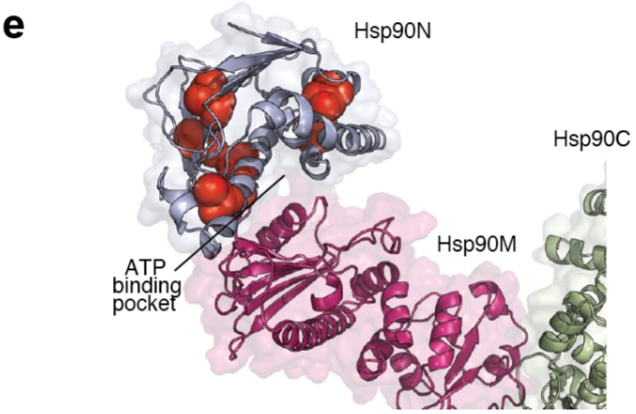

Supplementary Figure 1. Influence of nucleotides on the

Hsp90/FKBP51/Tau interaction. (**a**) SEC-MALS analysis shows that Hsp90 is a dimer in solution, while FKBP51 and the Hsp90NM construct (lacking the Hsp90C domain, which is responsible for dimerization) are monomeric. (**b**) SAXS radii of gyration (R_g) and maximum particle sizes (D_{max}) obtained for different Hsp90 conformations, co-chaperones and complexes. The increased dimensions of the Hsp90/FKBP51/Tau complex when compared to the Hsp90/FKBP51 complex, which are consistently observed in the presence and absence of nucleotides, supports formation of the ternary chaperone/co-chaperone/client complex. (**c,d**) Differences in FKBP51-induced attenuation of Hsp90 Ile-methyl peak intensities ((I/I_0)) in the absence and presence of ADP (c) and AMP.PNP (d). Threshold lines indicate an arbitrary value of +/-0.2 difference in intensity. 80 μM proteins and 2 mM of the corresponding nucleotide were used. (**e**) Mapping of intensity differences shown in (d) on the open Hsp90 dimer structure. Consistent with nucleotide binding to the N-terminal domain of Hsp90, AMP.PNP-related intensity changes (spheres) were observed mostly in proximity to the ATP-binding pocket in the N-terminal domain of Hsp90.

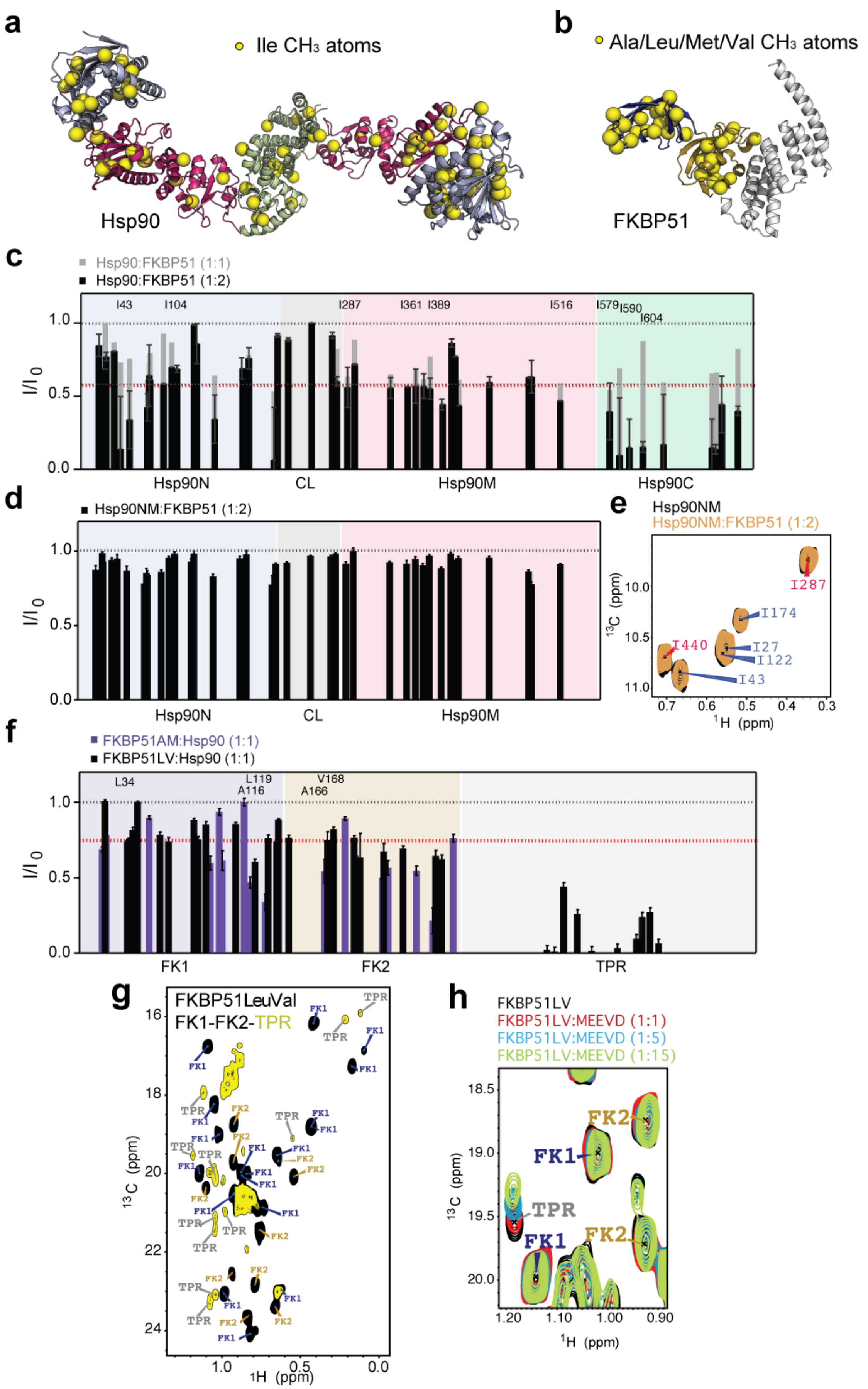

a
Ile CH3 atoms
Hsp90
b
Ala/Leu/Met/Val CH3 atoms
FKBP51
c
Hsp90:FKBP51 (1:1)
Hsp90:FKBP51 (1:2)
I/I0
I43
I104
I287
I361
I389
I516
I579
I590
I604
Hsp90N
CL
Hsp90M
Hsp90C
d
Hsp90NM:FKBP51 (1:2)
e
Hsp90NM
Hsp90NM:FKBP51 (1:2)
I287
I174
I440
I27
I122
I43
13C (ppm)
1H (ppm)
f
FKBP51AM:Hsp90 (1:1)
FKBP51LV:Hsp90 (1:1)
L34
L119
A116
V168
A166
FK1
FK2
TPR
g
FKBP51LeuVal
FK1-FK2-TPR
h
FKBP51LV
FKBP51LV:MEEVD (1:1)
FKBP51LV:MEEVD (1:5)
FKBP51LV:MEEVD (1:15)

Supplementary Figure 2. NMR analysis of the Hsp90/FKBP51 interaction. (**a**) Distribution of NMR-assigned isoleucine methyl groups (yellow spheres) on the structure of extended, full-length human Hsp90 (PDB id: 5fwk). (**b**) Distribution of NMR-assigned alanine/methionine/leucine/valine methyl groups (yellow spheres) on the structure of full-length human FKBP51 (PDB id: 1kt0). (**c**) Residue-specific attenuation of isoleucine methyl signals of Hsp90 (80 µM) upon addition of increasing FKBP51 concentrations. I_0 and I are Hsp90 signal intensities in the absence and presence of FKBP51, respectively. (**d**) Same as (c), but using Hsp90NM (100 µM) instead of full-length Hsp90. In agreement with the dependence of Hsp90/FKBP51 complex formation on the interaction between the MEEVD C-terminal tail of Hsp90 and the TPR repeats of FKBP51 [1], cross peaks of a monomeric Hsp90NM construct, were not perturbed by addition of FKBP51. (**e**) Inset showing the same spectral region shown in (Fig. 1d), for comparison. Signals in the isolated Hsp90NM construct are unaffected in the presence of FKBP51, supporting the absence of interaction. (**f**) Analysis of leucine-valine (100 µM protein, black bars) and alanine-methionine (100 µM protein, purple bars) selectively-labeled FKBP51 binding to Hsp90. Residues of all domains are affected. (**g**) Methyl-TROSY [2] spectra of Leu/Val labeled FKBP51. Cross peaks corresponding to the TPR region are highlighted in yellow. (**h**) In order to unambiguously identify TPR-belonging moieties, we performed NMR titrations of Leu/Val labeled FKBP51, using increasing amount of a synthetic peptide containing MEEVD, *i.e.* Hsp90 C-terminal characteristic sequence (Fig. 1e). Up to ten Leu/Val cross peaks showed chemical shift perturbation induced by binding to the MEEVD peptide, fully consistent with the recent crystallographic structure of the FKBP51/MEEVD complex [3]. These specific cross peaks were used to illustrate the signal perturbation from the TPR region induced upon binding to FKBP51 shown in (f) and in (Fig. 2e, 2g and 3d). Because of the lack of sequence-specific assignments, they were randomly located at positions of Leu/Val residues present in the MEEVD binding site as seen in the crystal structure (PDB id: 5njx). In (c) and (f), labels indicate residues located on the structures in figures 1e and 2f. Red dotted lines represent twice the standard deviation of the averaged observable signal perturbation and are used as a threshold to highlight residues in figures 1e and 2f. Error bars in (c),

(d), and (f) were calculated based on NMR signal-to-noise ratio.

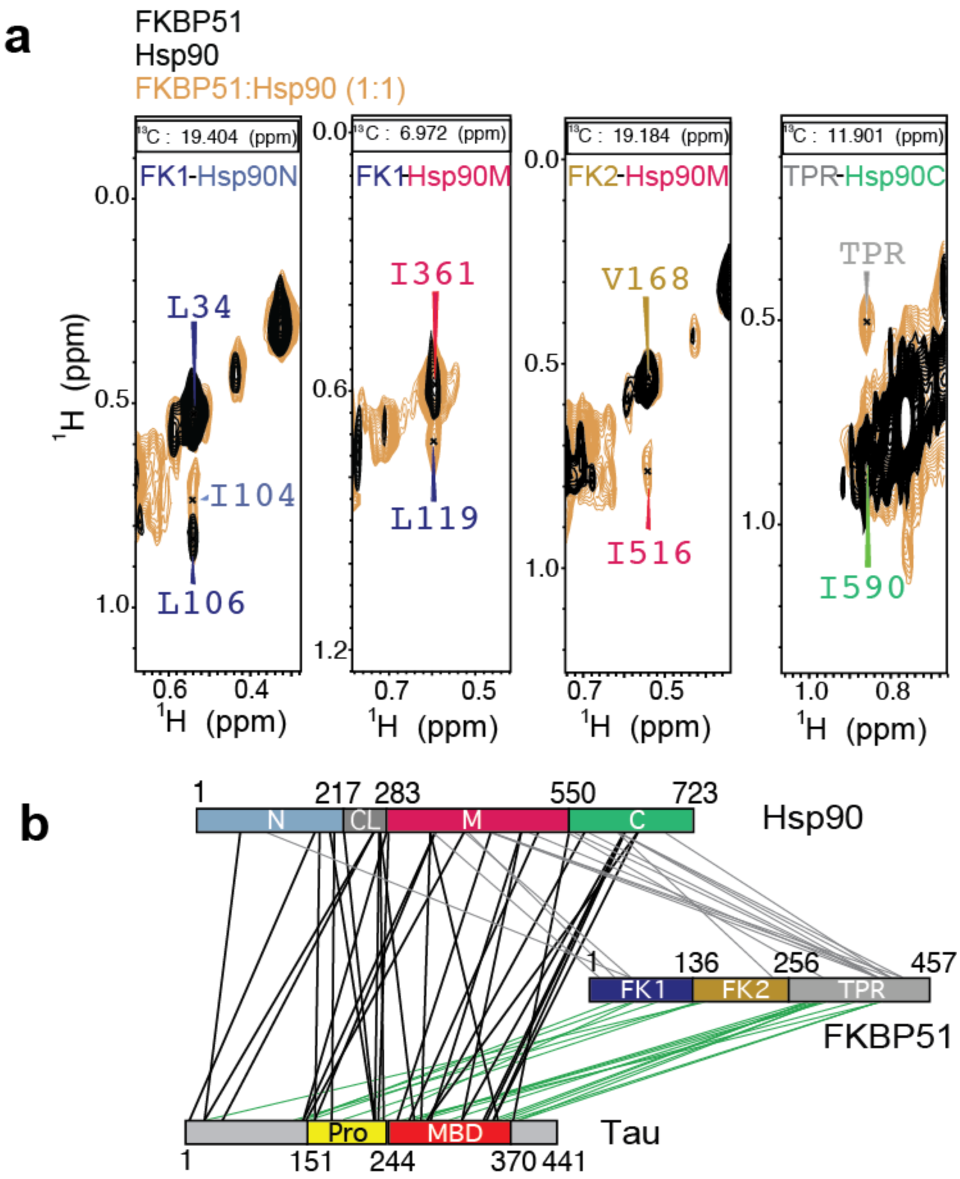

Supplementary Figure 3. Intermolecular contacts in the Hsp90/FKBP51 and Hsp90/FKBP51/Tau complex. (**a**) Superposition of selected regions from 3D ^{1}H-^{13}C-^{1}H NOESY spectra of the isolated Hsp90 and FKBP51 proteins (in black) and the complex (in orange). Intermolecular NOE peaks are only present in the mixture (orange). For comparison, the intramolecular NOE peak between FKBP51's Leu34-Leu106 is shown (in black, left panel). (**b**) Schematic representation of intermolecular cross-links found in the ternary Hsp90/FKBP51/Tau complex by XL-MS (Supplementary Table 2-4).

a

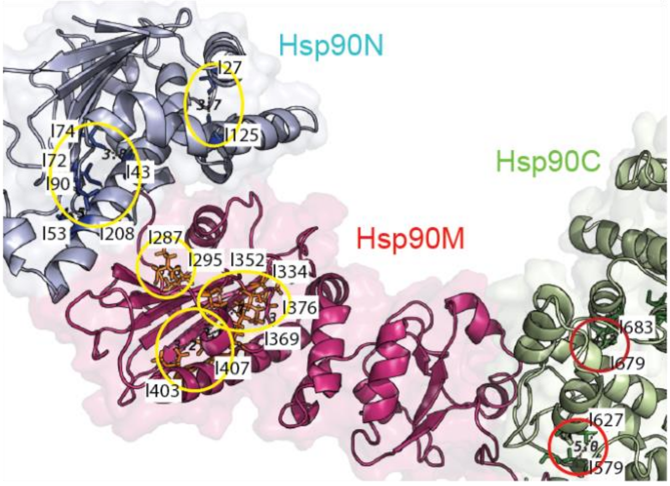

Hsp90 intramolecular NOEs

NOE	DOMAIN	OBSERVED
27-122	N	overlap
27-125	N	✔
43-72	N	overlap
43-74	N	✔
43-212	N-CL	overlap
53-90	N	✔
72-208	N	✔
72-212	N-CL	overlap
208-212	N-CL	overlap
287-295	M	✔
334-376	M	✔
352-369	M	✔
352-407	M	✔
369-407	M	✔
403-407	M	✔
579-627	C	✔
679-683	C	✔

b

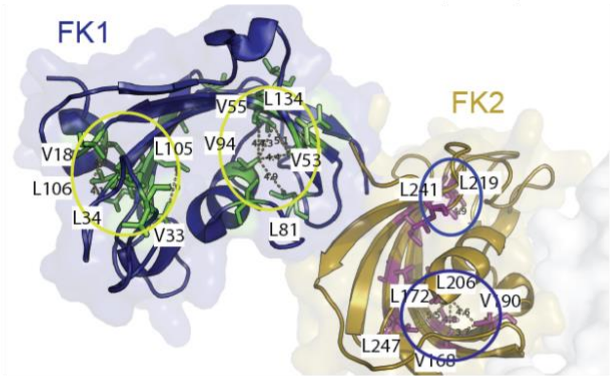

FKBP51 intramolecular NOEs

NOE	DOMAIN	OBSERVED
18-106	FK1	✔
33-105	FK1	✔
34-106	FK1	✔
37-40	FK1	overlap
53-94	FK1	✔
55-94	FK1	✔
61-128	FK1	overlap
81-94	FK1	✔
53-134	FK1	✔
94-134	FK1	✔
166-253	FK2	✔
168-190	FK2	✔
168-206	FK2	✔
168-247	FK2	overlap
172-186	FK2	✔
172-245	FK2	overlap
190-206	FK2	✔
206-245	FK2	overlap
206-247	FK2	✔
219-241	FK2	✔

c

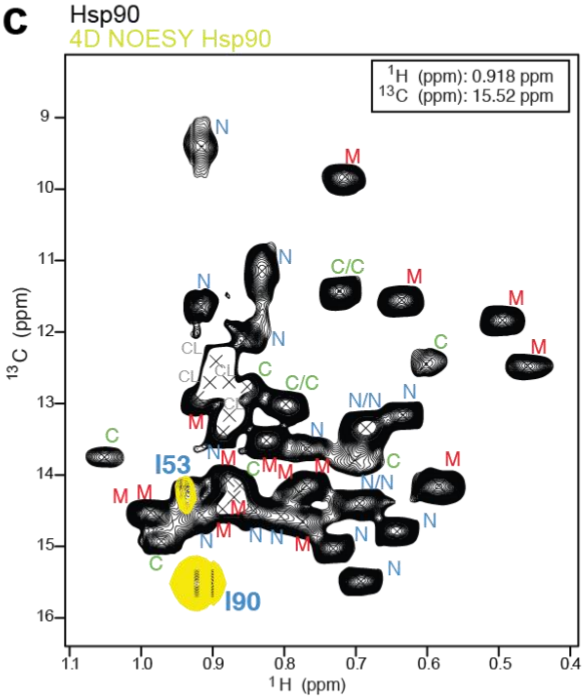

Supplementary Figure 4. The internal structure of the domains of Hsp90

and FKBP51 is retained in the Hsp90/FKBP51 complex. (**a,b**) Experimentally observed intramolecular NOEs in Hsp90 (a) and FKBP51 (b) within the 280 kDa Hsp90/FKBP51 complex. The two tables show all possible methyl NOE contacts according to the atomic structures of Hsp90 (PDB code 5fwk) and FKBP51 (PDB code 1kt0). All NOEs, which are predicted on the basis of the 3D structures, were present in the spectra, indicating that the internal structure of the domains was retained in the complex. Residues, for which intramolecular NOEs were detected, are highlighted in the 3D structures of Hsp90 (a) and FKBP51 (b) and are found in all the domains. (**c**) Overlay of the 2D methyl-TROSY spectrum of Hsp90 (black) with the projection of the plane corresponding to Ile90 from the 4D ^{1}H-^{13}C-^{13}C-^{1}H NOESY[4] experiment (yellow). A strong specific NOE contact between Ile90 (strong diagonal peak) and Ile53 can be observed, which corresponds to a distance of 4.5 Å between both methyl side-chain groups in the atomic structure (a).

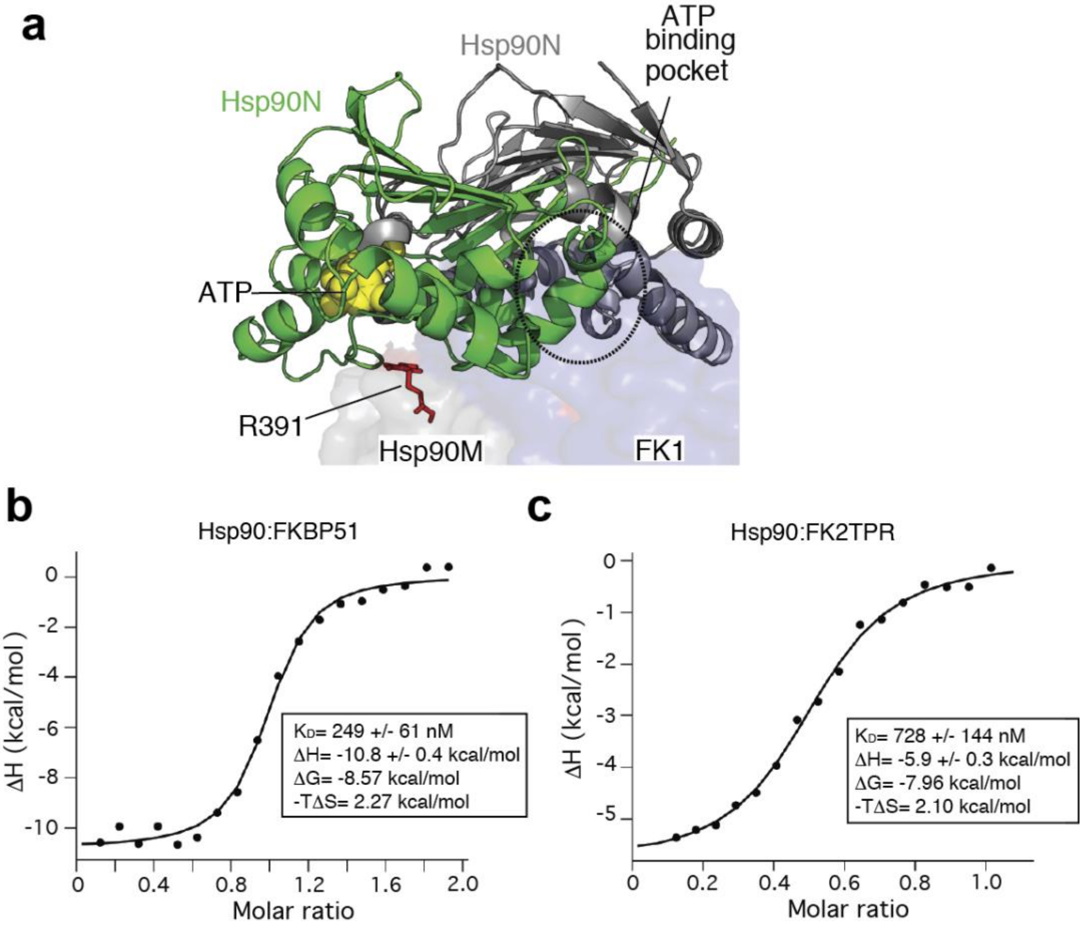

Supplementary Figure 5. Rotation of Hsp90N in the Hsp90/FKBP51 complex. (**a**) Through rotation of Hsp90N (in grey and green, PDB id: 5fwk) the ATP binding site moves away from R391. (**b,c**) ITC of FKBP51 (b) and FK2-TPR (c) binding to Hsp90 and the energetics of binding. Of note is the increase in enthalpy of binding for the interaction of FKBP51 and Hsp90 compared to FK2-TPR and Hsp90 interaction, reflecting the larger interacting surface for full-length FKBP51 binding.

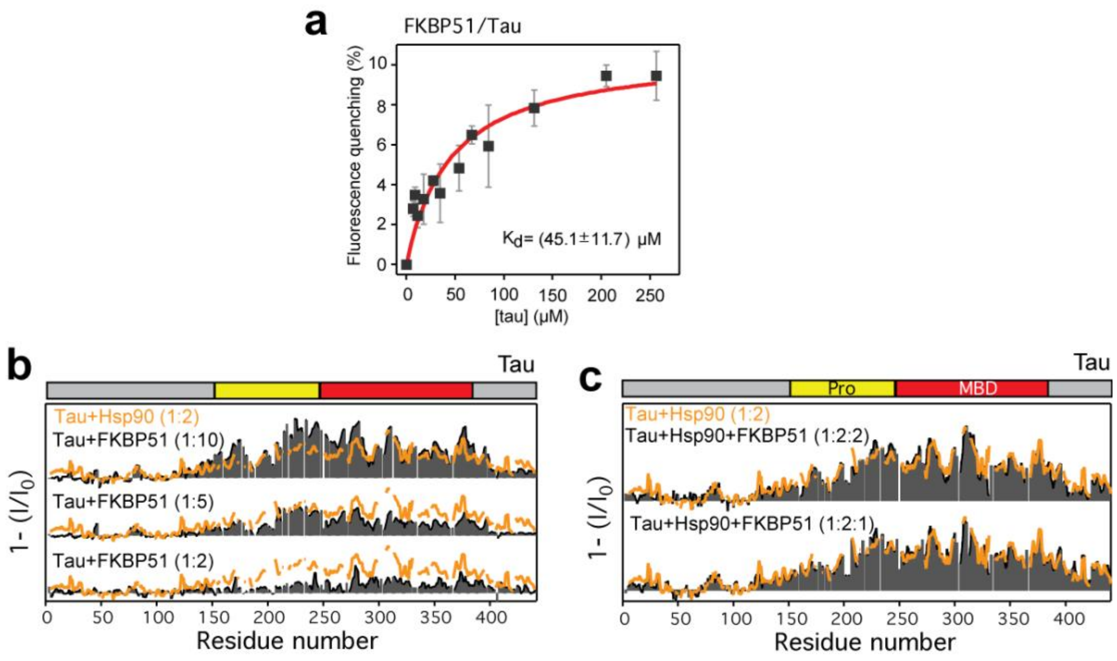

Supplementary Figure 6. Tau binds to FKBP51 with lower affinity in the absence of Hsp90. (**a**) Fluorescence affinity measurements of FKBP51/Tau interaction. The error was calculated for each Tau concentration using standard deviation of three independent experiments (**b**) NMR titration experiments of Tau binding to increasing concentrations of FKBP51 (from bottom to top). In orange line we show the Tau/Hsp90 (1:2) binding profile, supporting that Tau employs identical regions to bind to FKBP51, but with lower affinity. (**c**) The addition of increasing amounts of FKBP51 in the ternary complex does not affect Tau binding, indicating that Tau binds preferentially to Hsp90 in the ternary complex. For comparison, Tau/Hsp90 (1:2) binding profile is shown.

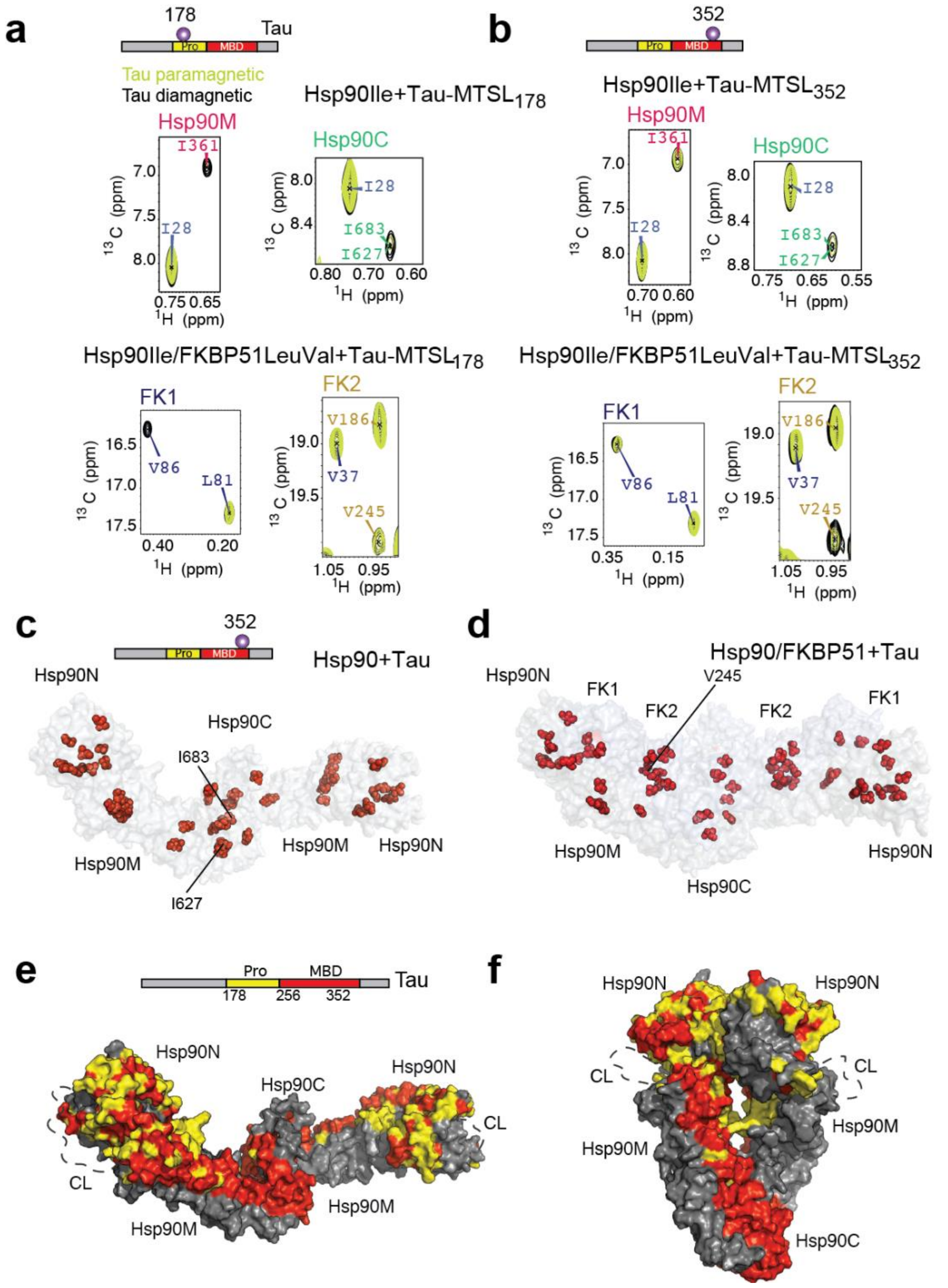

Supplementary Figure 7. PREs show the head-to-head arrangement in the Hsp90/Tau complex and the clustering of Tau's proline-rich region induced by FKBP51. (**a**,**b**) Superposition of selected regions from methyl TROSY spectra of Hsp90 (top) and FKBP51 (present in the Hsp90/FKBP51 complex, bottom) in presence of paramagnetic (green) and diamagnetic (black)

Tau showing specific PREs caused by the single spin-labeled Tau samples at position 178 (a) and 352 (b). In the Hsp90/Tau complex (top), a single spin-labeled Tau can induce PREs on very distant regions of the Hsp90 structure (178-labeled Tau induced PREs on Ile361, Ile627 and Ile683), which in turn are different from PREs caused by 352-tagged Tau (b). Therefore, despite being highly promiscuous, the specificity of the interaction allows for PRE- and XL-MS-based (Supplementary Table 3-5) interaction mapping (Fig. 5b). In contrast, only Tau tagged at position 178 induces PREs on FKBP51's Val86 (located on FK1, (a)), while only 352-tagged Tau induce PREs on FKBP51's Val245 (located on FK2, (b)), indicating that FKBP51 changes the conformational landscape of Tau towards a clustering of the proline-rich region around Hsp90N and FK1 (Fig. 5d). (**c,d**) Residues showing PREs (red spheres) from MTSL-tagged Tau at position 352 are highlighted on the structures of Hsp90 (c) and the Hsp90/FKBP51 complex (d). The PREs for 352-tagged Tau samples are observed in all Hsp90 and FKBP51 domains. The attachment site of the paramagnetic MTSL-tag in Tau (residues 178 and 352) is schematically shown above. For clarity, 256-tagged Tau is not shown. Domain-specific PREs are listed in Supplementary Table 5. (**e,f**) PRE-based mapping (containing PREs obtained from 178-, 256- and 352-tagged Tau) of Tau binding to the extended (e) and closed (f) Hsp90 dimer (PDB id: 5fwk), suggesting that Hsp90 can open and close with Tau bound. Yellow area corresponds to the binding interface of Tau's proline-rich region and red to Tau's microtubule-binding domain (Fig. 5b). Samples contained 30 μM of Tau and Tau:Hsp90 and Tau:Hsp90:FKBP51 molar ratios of 1:2 and 1:2:2, respectively.

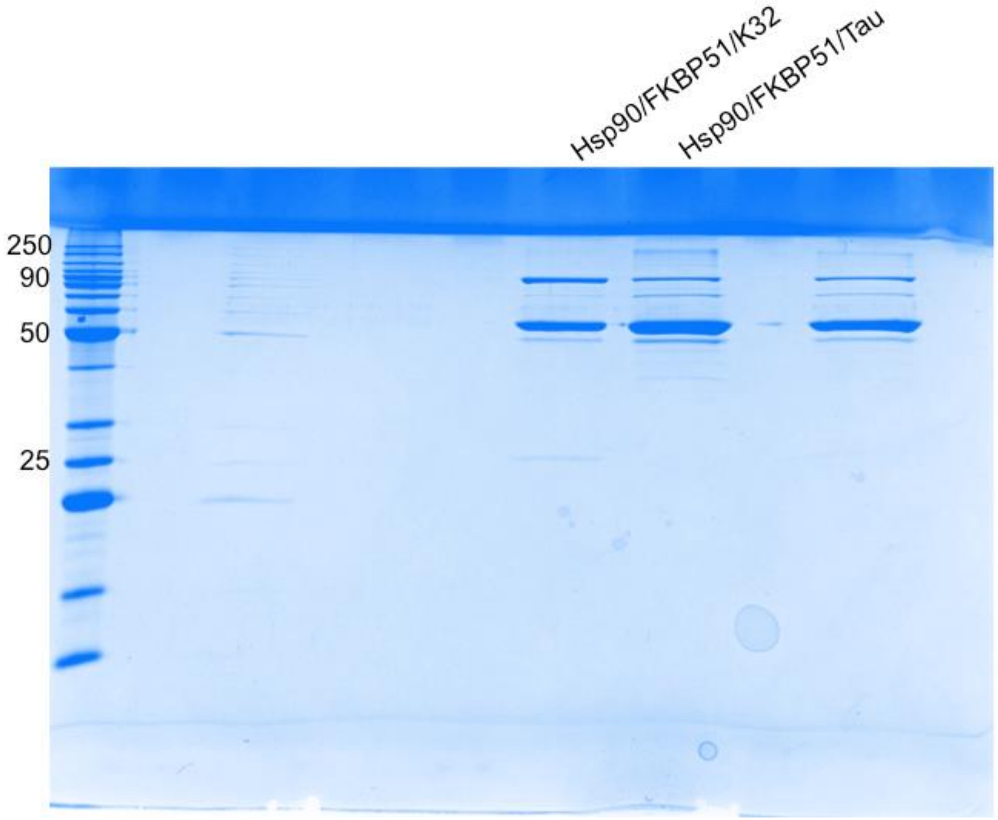

Supplementary Figure 8. SDS-PAGE of the SEC-MALS elution fractions highlighted in Figure 4c.

Supplementary Table 1. Hsp90/FKBP51 intermolecular NOEs in the complex. Only NOE contacts between methyl groups belonging to well-structured regions of the proteins are listed.

NOE	DOMAINS
I104-L34	Hsp90N-FK1
I276-L119	Hsp90CL-FK1
I361-L119	Hsp90M-FK1
I516-A166	Hsp90M-FK2
I516-V168	Hsp90M-FK2
I579-TPR	Hsp90C-TPR
I590-TPR	Hsp90C-TPR

Supplementary Table 2. Hsp90/FKBP51 intermolecular cross-links.

Residue in Hsp90	Residue in FKBP51	DOMAINS
106	60	Hsp90N-FK1
353	8	Hsp90M-FK1
401	60	Hsp90M-FK1
405	38	Hsp90M-FK1
434	385	Hsp90M-TPR
434	399	Hsp90M-TPR
434	415	Hsp90M-TPR
490	385	Hsp90M-TPR
550	307	Hsp90C-TPR
556	385	Hsp90C-TPR
576	385	Hsp90C-TPR
622	230	Hsp90C-FK2
623	385	Hsp90C-TPR
623	399	Hsp90C-TPR
623	415	Hsp90C-TPR
684	399	Hsp90C-TPR

Supplementary Table 3. Hsp90/Tau Intermolecular Cross-links

Residue in Hsp90	Residue in Tau	DOMAINS
63	24	Hsp90N-Nterm
179	2	Hsp90N-Nterm
185	163	Hsp90N-Pro
198	225	Hsp90N-Pro
203	180	Hsp90N-Pro
218	225	Hsp90CL-Pro
264	24	Hsp90CL-Nterm
264	44	Hsp90CL-Nterm

274	225	Hsp90CL-Pro
274	240	Hsp90CL-Pro
274	274	Hsp90CL-MBD
283	148	Hsp90M-Nterm
285	234	Hsp90M-Pro
346	290	Hsp90M-MBD
346	385	Hsp90M-Cterm
353	150	Hsp90M-Nterm
353	163	Hsp90M-Pro
398	150	Hsp90M-Nterm
434	257	Hsp90M-MBD
480	298	Hsp90M-MBD
480	331	Hsp90M-MBD
504	267	Hsp90M-MBD
551	395	Hsp90C-Cterm
572	294	Hsp90C-MBD
622	347	Hsp90C-MBD
623	331	Hsp90C-MBD
623	353	Hsp90C-MBD
623	370	Hsp90C-MBD
648	290	Hsp90C-MBD
648	370	Hsp90C-MBD

Supplementary Table 4. Intermolecular cross-links found in the ternary Hsp90/FKBP51/Tau complex.

Residue in FKBP51	Residue in Tau	DOMAINS
28	150	FK1-Nterm
58	143	FK1-Nterm
60	225	FK1-Pro
88	150	FK1-Nterm
153	140	FK2-Nterm
155	24	FK2-Nterm
155	190	FK2-Pro
272	290	TPR-MBD
272	369	TPR-MBD
274	370	TPR-MBD
275	274	TPR-MBD
280	274	TPR-MBD
283	317	TPR-MBD
283	375	TPR-Cterm
283	395	TPR-Cterm
352	281	TPR-MBD
385	257	TPR-MBD
385	383	TPR-Cterm
385	395	TPR-Cterm

Supplementary Table 5. Hsp90 and Hsp90/FKBP51 residues experiencing PRE broadening induced by spin-labeled Tau.

Complex	Domain	178	256	352
Hsp90/Tau	Hsp90N	20, 27, 37, 90, 98, 122, 125, 174, 208	20, 28, 37, 43, 90, 98, 104, 174	27, 37, 43, 72, 74, 90, 98, 122, 181
	Hsp90-CL	212, 249, 271, 276	212, 224, 249	-
	Hsp90M	352, 361, 369, 389, 399, 403, 440	334, 361, 369, 389, 399, 403, 440	334, 352, 376, 399, 403, 407, 482, 485
	Hsp90C	569, 604, 627, 683	569, 579, 604, 627, 683	569, 579, 590, 604, 627, 633, 683
Hsp90/FKBP51/Tau	Hsp90N	27, 37, 43, 74, 75, 98, 104, 208	20, 27, 43, 74, 98, 122, 125, 174	27, 37, 43, 72, 74, 90, 98, 122, 181
	Hsp90-CL	249	224, 271, 276	-
	Hsp90M	389, 399	295, 334, 352, 361, 369, 389, 440, 482	352, 376, 389, 403, 407, 440
	Hsp90C	-	579, 590, 604, 627, 679, 683	579, 590, 604, 627, 683
	FK1	55, 86	40, 55, 86	18
	FK2	-	172, 186, 206, 219, 245, 247	142, 168, 172, 186, 190, 206, 219, 245, 247

Supplementary Table 6. HADDOCK docking statistics of the refined structure of the Hsp90/FKBP51 complex.

HADDOCK score	-89.0 +/- 6.1
RMSD from the overall lowest-energy structure	0.3 +/- 0.2
Van der Waals energy	-71.4 +/- 5.4
Electrostatic energy	-406.9 +/- 55.4
Desolvation energy	63.3 +/- 5.3
Restrains violation energy	4.3 +/- 0.24
Buried Surface Area	2540.6 +/- 73.8

Supplementary Table 7. Oligonucleotides used for cloning.

pET28/EcoRI/Hsp90N 5´	CATATGGAATTCATGCCTGAGGAAG
pET28/XhoI/Hsp90N 3´	CTTTATCTCTCGAG*CTACTA*CTCACCTTTCTCTTCC
pET28/NheI/Hsp90NM 5´	CATATGGCTAGCATGCCTGAGGAAG
pET28/BamHI/Hsp90NM 3´	CCTTGCTGGATCC*CTACTA*CTTCTTCTCC
pET28/NheI/Hsp90M 5'	CAGAGGCTAGCAAAGGTGAGAAAGAAGAGG
pET28/BamHI/Hsp90M 3´	CTTCGGATCCTTACTTCTTCTCCTCCTCATCC
pMAL/BamHI/Hsp90C 5´	CAGGGGATCCAAGATGGAAGAGAGCAAGGC
pMAL/HindIII/Hsp90C 3'	GCAGAAGCTTCTAATCGACTTCTTCCATGCGAGACGCAT
pET28/EcoRI/FK1 5'	GATCCGAATTCATGACTACTGATGAAGG
pET28/HindIII/FK2 3'	GGTATCAAGCTT*CTACTA*TTCTTTGGCCTTTTCG
pET28/EcoRI/FK2 5'	GCTGAATTCTTCAAAGGAGAGGATTTATTTG
pET28/BamHI/TPR 3'	GTTGGATTCTCATACGTGGCCCTCAGG

Supplementary References

1. Radanyi, C., Chambraud, B. & Baulieu, E.E. The ability of the immunophilin FKBP59-HBI to interact with the 90-kDa heat shock protein is encoded by its tetratricopeptide repeat domain. *Proc Natl Acad Sci U S A* **91**, 11197-201 (1994).
2. Tugarinov, V., Hwang, P.M., Ollerenshaw, J.E. & Kay, L.E. Cross-Correlated Relaxation Enhanced 1H−13C NMR Spectroscopy of Methyl Groups in Very High Molecular Weight Proteins and Protein Complexes. *J Am Chem Soc* **125**, 10420-8 (2003).
3. Kumar, R., Moche, M., Winblad, B. & Pavlov, P.F. Combined x-ray crystallography and computational modeling approach to investigate the Hsp90 C-terminal peptide binding to FKBP51. *Sci Rep* **7**, 14288 (2017).
4. Tugarinov, V., Kay, L.E., Ibraghimov, I. & Orekhov, V.Y. High-resolution four-dimensional ^{1}H-^{13}C NOE spectroscopy using methyl-TROSY, sparse data acquisition, and multidimensional decomposition. *J Am Chem Soc* **127**, 2767-75 (2005).
